# Developmental feedbacks and the emergence of individuality

**DOI:** 10.1098/rsos.221189

**Published:** 2022-11-30

**Authors:** Sean M. Ehlman, Ulrike Scherer, Max Wolf

**Affiliations:** ^1^ SCIoI Excellence Cluster, Berlin, Germany; ^2^ Humboldt University, Berlin, Germany; ^3^ IGB – Leibniz Institute of Freshwater Ecology and Inland Fisheries, Berlin, Germany

**Keywords:** development, feedback loops, individual variation, state-dependent behaviour, fitness landscape

## Abstract

Behavioural individuality is a hallmark of animal life, with major consequences for fitness, ecology, and evolution. One of the most widely invoked explanations for this variation is that feedback loops between an animal's behaviour and its state (e.g. physiology, informational state, social rank, etc.) trigger and shape the development of individuality. Despite their often-cited importance, however, little is known about the ultimate causes of such feedbacks. Expanding on a previously employed model of adaptive behavioural development under uncertainty, we find that (i) behaviour-state feedbacks emerge as a direct consequence of adaptive behavioural development in particular selective environments and (ii) that the sign of these feedbacks, and thus the consequences for the development of behavioural individuality, can be directly predicted by the shape of the fitness function, with increasing fitness benefits giving rise to positive feedbacks and trait divergence and decreasing fitness benefits leading to negative feedbacks and trait convergence. Our findings provide a testable explanatory framework for the emergence of developmental feedbacks driving individuality and suggest that such feedbacks and their associated patterns of behavioural diversity are a direct consequence of adaptive behavioural development in particular selective environments.

## 1. Introduction

Behavioural individuality is a ubiquitous feature of animal populations [1–4], and feedback loops between behaviour and state (e.g. physiology, energetic state, informational state, social rank) are considered to be one of the key factors triggering and shaping the development of individuality [5–7]. The key intuition is that, during development, differential expression of behaviour between individuals causes differences in state which, in turn, favour behavioural responses that stabilize or even increase initial behavioural differences (positive feedbacks) or result in behavioural convergence (negative feedbacks) among individuals. Thus, it is often argued that (i) in the presence of positive feedbacks, even small initial differences between individuals may give rise to long-lasting and substantially enhanced among-individual differences and (ii) in the presence of negative feedbacks, behavioural differences tend to be transitory, converging to a common value.

While behaviour-state feedback loops are widely cited as one possible explanation for behavioural individuality [8–15] (but see [16] for further discussion on alternative, non-mutually exclusive causes of behavioural individuality), at present, very little is known about two fundamental questions associated with feedbacks. First, what ultimately causes behaviour-state feedbacks? In particular, can behaviour-state feedbacks be understood (as typically suggested; [5,7]) as an outcome of adaptive state-dependent behaviour in particular ecological conditions? If so, can we delineate the conditions that promote these feedbacks? Second, can we detect generic mechanisms that promote feedbacks between a potentially large number of behaviour-state combinations in a range of different contexts, or are feedbacks limited to only very specific behaviour-state combinations in specific situations?

Behaviour-state feedbacks unfold dynamically over ontogeny, making mathematical models an essential and powerful approach to generate basic insights and testable predictions guiding empirical research. The few previous modelling approaches have focused on specific behaviour-state combinations (e.g. foraging effort and safety; [17]), assuming a direct link between them [5,7]. While such models provide important proof-of-concept, our goal here is to provide an analysis of the emergence of behaviour-state feedbacks (i.e. without first assuming specific feedbacks) and its individual- and population-level consequences. In order to do so, we focus on a central aspect in the development of behaviour: the interaction between that behaviour, an incrementally unfolding underlying state, and the selective environment (i.e. the shape of the fitness landscape associated with an animal's state).

## 2. Model

Our goal is to investigate the interaction between a sequentially repeated behavioural choice, an underlying state that is incrementally unfolding over ontogeny, and the selective environment. In order to do so, we employ a modelling framework of incremental behavioural development under uncertainty first presented by Frankenhuis & Panchanathan to investigate optimal cue sampling through development [18], and later expanded to explore the evolution of sensitive periods [19] and examine the effects of variable cue reliabilities [20] or fluctuating environments [21]. We briefly describe the current model here and provide further details in electronic supplementary material, Appendices 1 and 2.

Each individual experiences one of two possible environmental conditions, *E*_0_ or *E*_1_, through the entirety of development. Individuals must incrementally develop a behavioural phenotype: in each developmental timestep, individuals develop in either of two phenotypic directions *y*_0_ or *y*_1_ where, for simplicity, we assume that increments in the direction of *y*_0_ only yield a fitness benefit in environment *E*_0_ and increments in the direction of *y*_1_ only yield a fitness benefit in environment *E*_1_. While individuals cannot observe the condition of the environment directly, in each developmental timestep, individuals receive an imperfect cue that signals the condition of the environment. After receiving that cue, individuals update their estimate of the current environmental condition and then decide between taking one of two behavioural actions, *y*_0_ or *y*_1_. Each behavioural choice, in turn, incrementally contributes to a developing specialization state, which corresponds to the total number of previous behavioural actions in either of the two phenotypic directions *y*_0_ and *y*_1_. A fitness function relating the value of this state at the end of development to fitness (in combination with the environmental condition) is then used (via stochastic dynamic programming [22]) to determine the optimal behaviour choice at each timestep through development.

We thus investigate the repeated choice between two behavioural actions through development under environmental uncertainty where behavioural choices contribute to an individual's incrementally developing state. Viewed in the context of behaviour-state connections, ‘behaviour’ thus corresponds to an individual's choice at any given timestep, and ‘state’ to the total number of phenotypic increments accumulated in either of the two phenotypic directions by that timestep. To be concrete, one might think that the two behavioural options correspond two alternative foraging tactics and the state variable to the experience (or skill) that an individual has accumulated with each of the two tactics. In this case, the subject has a choice of two behaviours (foraging tactic A or B) at each timestep, and an individual's state at a given timestep is their cumulative skill with each of these foraging tactics up to that timestep.

At any given timestep, the optimal choice of an individual will depend on its current belief about its environmental condition, corresponding to a posterior estimate obtained from the Bayesian integration of cues received up to that point (see electronic supplementary material, Appendix 1). Importantly, the optimal behaviour of an individual may also depend on its previous behaviours, potentially producing a mechanism underlying feedback loops: as accelerating fitness functions (i.e. relating accumulated state to fitness) represent increasingly larger returns associated with behavioural specialization, the more an individual has specialized, the more beneficial further specialization may become, thus potentially producing a positive feedback loop and behavioural divergence. Likewise, as decelerating fitness functions give rise to continuously smaller returns to specialization, such fitness landscapes may result in a scenario whereby the more an individual has specialized, the less beneficial further specialization becomes, potentially promoting negative feedback loops and behavioural convergence. In order to investigate this basic intuition, we examine three basic fitness landscapes relating the number of correctly expressed behaviours at the end of development to fitness: (1) concave (i.e. decelerating), (2) convex (i.e. accelerating) and (3) linear (i.e. constant) fitness functions (see electronic supplementary material, Appendix 2). Throughout, we use stochastic dynamic programming [22] to calculate optimal developmental programs; we then simulate populations of individuals following these programs, where all individuals start with a prior of 0.5 of being in either of the two environments and proceed through development for 20 timesteps, receiving cues and updating priors at each timestep. Cue reliability (i.e. the probability of receiving cue *c*_0_ when in environment *E*_0_, and the probability of receiving cue *c*_1_ when in environment *E*_1_) for all figures shown in the main text is set at 0.55, and results for alternative cue reliabilities are given in the appendices and discussed throughout the main text. All code is written in Python 3 and available on Github (https://github.com/smehlman/Developmental_feedbacks).

## 3. Results

**Figure 1.**
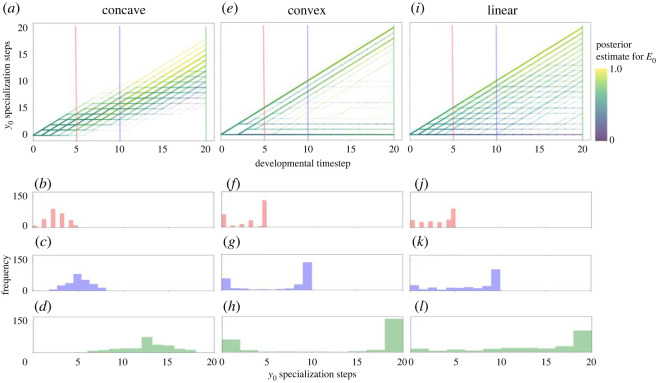
Optimal developmental trajectories of a simulated population of 200 individuals in an *E*_0_ environment, where populations are faced with a concave, convex, or linear fitness landscape, respectively (*a*, *e*, *i*). Histograms show the spread of individual variation at timepoints corresponding to the vertical, coloured lines in the trajectory plots: early (5 timesteps; red line), midway through (10 timesteps; blue line), and late (20 timesteps; green line) in development. Line segments along an individual's developmental trajectory are coloured by an individual's posterior probability of being in *E*_0_. As can be seen, concave fitness landscapes lead to frequent ‘switching’ between behavioural choices (*a*), promoting populations with Gaussian-like distribution of phenotypic states around a population mean (*b*–*d*) with spread around this mean mediated primarily by stochastic differences in cues received (i.e. posterior estimates). In sharp contrast, convex fitness landscapes lead to early behavioural specialization (*e*) and bimodal phenotypic distributions (*f–h*), even when beliefs about the state of the environment (i.e. posterior estimates) are quite similar. Linear fitness landscapes result in an intermediate pattern of behavioural specialization (*i*) with right-skewed phenotypic distributions (*j–l*). Cue reliability is 0.55.

Inspecting our optimal developmental trajectories and their unfolding over developmental time, we find marked differences depending on the selective environment in which developmental programs have evolved, in line with previously observed patterns [18,19]. Populations evolving in concave fitness landscapes exhibit a Gaussian-like distribution around a population mean with approximately symmetrical—and relatively little—variation around the mean (figure 1*a–d*), with spread around this mean governed primarily by stochastic differences in posterior probability estimates (i.e. by differences in cues received). In sharp contrast, populations that have evolved in convex fitness landscapes tend to show an approximately bimodal phenotypic distribution (figure 1*e–h*), with some individuals having followed dramatically different life trajectories despite having relatively similar beliefs (i.e. posterior probability estimates) regarding the true condition of the environment. Linear fitness landscapes result in a heavily right-skewed phenotypic distribution (figure 1*i–l*) and a pattern whereby beliefs correspond roughly to phenotypic state (though this correspondence is not as strong as with populations evolved under concave fitness landscapes).

**Figure 2.**
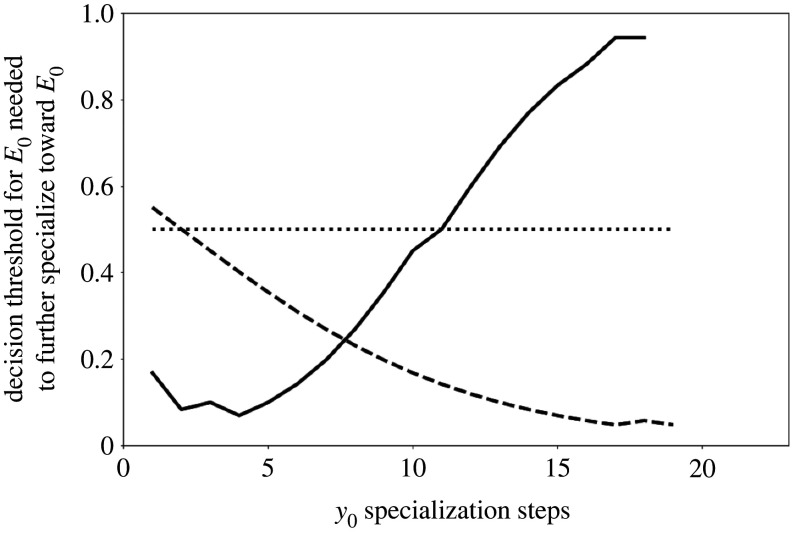
Curves representing the decision threshold (minimum threshold posterior estimate) values for choosing to specialize toward *E*_0_ as a function of the number of specialization steps already accrued in that direction (*y*_0_). The three curves correspond to thresholds for concave (solid), linear (dotted), and convex (dashed) fitness landscape. As can be seen, in a concave fitness landscape, this threshold increases with an increasing number of previous developmental steps in this direction, making further specialization in this direction less likely. By contrast, in a convex landscape, this threshold decreases with the number of previous specialization steps in this direction, making further specialization steps more likely. In linear fitness landscapes, individuals are not more or less likely to exhibit a particular choice given a certain number of previous specialization steps. Decision thresholds were computed from a simulation of a large population (*n* = 10,000), where cue reliability was 0.55.

In order to understand the developmental mechanisms that generate these marked differences, it is important to consider that concave fitness landscapes give rise to decreasing returns of specialization while convex landscapes give rise to increasing returns of specialization. In the case of concave fitness landscapes—conditional on there being sufficient uncertainty about the state of the environment (see below)—this leads to frequent ‘switching’ between behavioural choices, *y*_0_ and *y*_1_, as the expected benefit of a further developmental step in a particular direction decreases with previous choices in the direction. The solid line in figure 2 illustrates this: as individuals take more developmental steps in a particular phenotypic direction (e.g. accrue more experience with a particular behaviour), the threshold posterior probability, that is, the posterior probability above which individuals specialize further in this direction, increases, making further steps in this direction increasingly less likely. Such a pattern in which having a greater number of previous developmental steps in a particular direction decreases the probability that yet further phenotypic steps in that direction corresponds to a negative feedback between behaviour (i.e. the choice at any timepoint between *y*_0_ and *y*_1_) and state (i.e. the number of previous developmental steps in a particular direction accrued up to that timepoint). In sharp contrast, in convex fitness landscapes (figure 2, dashed line), this threshold posterior probability for further specialization decreases with the number of previous developmental steps in this direction, making further developmental steps in this direction more likely. This scenario, whereby amassing experience in a particular direction makes it more likely that an individual further develops their behavioural phenotype in the same direction in future timesteps, thus corresponds to a positive feedback between behaviour and state. These general patterns relating the development of states to ‘switching’ threshold hold true largely irrespective of cue reliability values (see electronic supplementary material, Appendix 4), but their effects on patterns of individual variation are particularly pronounced when cue reliability is relatively low. We also note that, as expected, in the case of linear fitness benefits (figure 2, dotted line), individuals are not any more or less likely to exhibit a particular choice given a certain degree of prior specialization. In this case, the bidirectional link between behaviour and state is effectively severed: while behaviour affects state, state (i.e. degree of experience/specialization already accrued) does not affect behaviour (i.e. the choice between *y*_0_ and *y*_1_ given a cue set).

**Figure 3.**
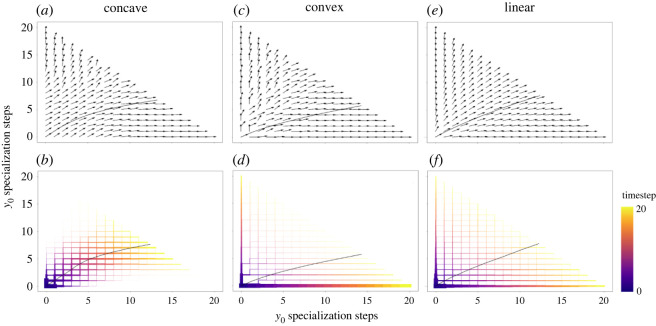
Phenotypic phase diagrams and developmental trajectories through phenotypic space in an *E*_0_ environment in which *y*_0_ increments are shown on the x-axis and *y*_1_ increments are shown on the y-axis. For all panels, development begins at point [0, 0]. (*a*, *c*, *e*) show phase diagrams for concave, convex, and linear fitness scenarios, respectively, in which developmental channeling is depicted by an arrow from each phenotypic state (*y*_0_, *y*_1_ combination); the angle of each arrow is proportional to the population frequency of achieving [*y*_0_ + 1, *y*_1_] versus [*y*_0_, *y*_1_ + 1] conditional on having achieved the phenotypic state of [*y*_0_, *y*_1_]. Black solid lines represent the mean phenotypic state in the population through development. (*b*, *d*, *f*) depict developmental trajectories in which line thickness corresponds to the population frequency in a particular phenotypic state and colour from purple to yellow represents the timestep in development from birth until the end of development. Again, black solid lines represent phenotypic averages in the population through development. Cue reliability is 0.55.

To better understand how positive and negative feedbacks emerging in convex and concave fitness scenarios, respectively, shape behavioural-experiential trajectories through development, we visualize a ‘phenotypic phase space’ (a sort of derived Waddingtonian ‘epigenetic landscape’ [23]) that depicts how individuals are channeled through phenotypic space (i.e. possible combinations of experiences with *y*_0_ and *y*_1_) from birth until the end of development (figure 3*a,c*,*e*). These phenotypic phase spaces depict the probability of being channeled in any direction through development conditional on being at any particular phenotypic state (i.e. the direction of each arrow is weighted by the probability of moving in any direction in phenotypic space): the differences in the ‘flow’ through these spaces dependent on the shape of the fitness functions visually represents the influence of the selective environment on the developmental program through which phenotypes are produced. In the scenario of negative feedbacks (i.e. concave fitness function), individuals in areas of phenotypic space farther away from the mean phenotypic value are systematically channeled toward the mean (figure 3*a*), resulting in a population frequency distribution centered relatively tightly around the population mean (figure 3*b*), explaining also the observed Gaussian-like distribution of individuals' behavioural-experiential phenotypes (figure 1*b–d*). We note that the phenotypic phase diagram of linear fitness scenarios also shows some amount of channeling toward the mean, though this channeling does not occur as early and is generally not as strong (figure 3*e*). Compared to both concave and linear cases, however, the phenotypic phase diagram for the convex fitness scenario is dramatically different: the developmental channeling tends to create divergence early in development and maintain this diverging force through most of the phenotypic space (figure 3*c*), resulting in a population frequency distribution with a steep ridge either side of which individuals are strongly shunted toward one or the other highly specialized state (figure 3*d*). This strong, maintained force driving phenotypic divergence throughout development helps explain the almost bimodal distribution of specialized phenotypes seen in figure 1*f–h*.

## 4. Discussion

We find that the optimal developmental unfolding of behavioural traits under environmental uncertainty leads to behaviour-state feedback loops. Until now, such feedbacks, while often invoked as a general explanation for the origin and maintenance of behavioural diversity through development, have only been investigated using specific, proof-of-concept models that focus on interactions between particular behaviour and state variables [5,7,17]; here, in contrast, we show how these feedbacks arise as a direct consequence of optimal developmental programs in particular selective environments. We suggest that, due to the ease of generating these feedbacks by simply modelling the optimal unfolding of traits under environmental uncertainty, such feedbacks may emerge as a direct consequence of the fitness landscapes that are defined by a species' particular ecology, and the sign of these feedbacks (positive versus negative) can be directly predicted from the shape of a species' fitness landscape. Indeed, in our model, such feedback loops always emerge except in the case of linear fitness landscapes.

In particular, we uncover a pattern whereby concave (i.e. diminishing returns) fitness landscapes give rise to negative behaviour-state feedbacks promoting behavioural convergence among individuals, while convex fitness landscapes give rise to positive feedbacks resulting in behavioural diversification among individuals. While it is clear from empirical studies that developmental processes are crucial in influencing the spread of within- and among-individual variation [24,25], our model produces novel results that implicate the specific shape of the selective environment in the adaptive developmental programs generating patterns of individual variation. That said, nonlinear fitness functions have previously been shown to have major effects on the development and evolution of a range of traits, including foraging behaviours [26], risk assessment and management [27,28], life-history transitions [29], the use of heuristics in decision making [30], and social behaviours [31,32]. A concave fitness function, for example, may underlie the evolution of certain cooperative behaviours such as bloodmeal sharing in vampire bats [33] or brood care in *Polistes* wasps [34]. In our model, we thus extended the importance of the specific relationship between fitness benefits and trait development to the realm of state-behaviour feedbacks and the generation and maintenance of individual behavioural variation.

While negative feedbacks in our model produce a population of relatively homogeneous and somewhat generalized individuals, positive feedbacks generate a nearly bimodal distribution of two types of highly specialized behavioural morphs. Interestingly, these two contrasting population types exhibit approximately the same mean degree of specialization: with relatively low cue reliability, both populations could broadly be considered as moderately generalized (versus specialized). This is directly comparable to previous insights that related individual variation to patterns of niche breadth on a population scale [35]: a population with a broad niche breadth may be composed of many similar generalists with individual niche breadth closely matching the population-level niche breadth, or alternatively, such a population may be composed of many specialists, each with narrow niches, but with significant variation among individuals in niches. It is now well-established that inter-individual trait variability—even comparing between populations with similar trait means—may affect patterns and processes such as range sizes and dispersal patterns [36,37], community structure [38], and ecological functioning [39–41]. Our model suggests that feedbacks arising from specific selective regimes represent a major avenue for how such variation emerges, with potentially far-reaching ecological and evolutionary consequences.

Our work also connects to literature on the evolution of behavioural skill, competence or expertise, which will often be an incrementally developing trait. Empirical work has shown that the integration of cues early in life is an important predictor of behavioural competence later in life [42]. Early life cues regarding the social environment (e.g. density, sex ratio, competitive ability), for example, may channel individuals into behavioural types, mating strategies or dominance relationships best suited for their adult environment [43,44]. To be concrete, through development, sequences of aggressive interactions between individuals for resources can hone skillful fighting ability, as the choice to engage in a fight or not positively or negatively contributes to future fighting ability [45–47]. In these scenarios, we predict that—whenever individuals can find themselves in different environmental conditions in which the relative fitness benefits of developing toward different phenotypes differ (e.g. environments with contrasting densities favouring either a ‘hawk’ or ‘dove’ phenotype [48])—the shape of the fitness function is directly linked to the emergent pattern of variation. As we have seen above, skills that require less of an investment in expertise (experience) in order to achieve a relatively high payoff are likely to select for more homogeneous populations of intermediately specialized individuals, whereas those requiring high levels of experience in order to gain appreciable fitness benefits are likely to produce greater (bimodal) individual variation (e.g. ‘hawks' versus ‘doves' in aggressive interactions, ‘sneakers’ versus ‘displayers’ in mating interactions, etc.). Future modelling work that allows, for example, for reversible phenotypes or variable sizes of incremental phenotypic ‘steps’ available to developing organisms will be useful in determining the full range of scenarios in which feedbacks are likely to be most influential as a driver of individuality.

We have employed a model of incremental behavioural development to study the emergence of behaviour-state feedbacks and their consequences for patterns of inter-individual variability. This general framework of representing phenotypic development (as well as other similar frameworks; [49,50]) is powerful in its ability to uncover high-level processes such as the nature of behaviour-state feedbacks, the evolution of sensitive periods in development [19], trade-offs between specializing or environmental sampling [18], etc. Our findings are in line with previous findings linking environmental uncertainty and fitness function shapes to patterns of individuality at the end of development (fig. 3 in [18] and supplemental material in [19]). Our work adds to these findings by identifying the behaviour-state feedback mechanisms that are present in adaptive developmental trajectories and thus produce these specific patterns of individuality. Interestingly, while these feedback mechanisms are present across a wide range of cue reliabilities (electronic supplementary material, Appendix 4), the power of these feedbacks to produce distinct patterns of individual variation over the course of development (i.e. bimodal distribution in the case of positive feedbacks and unimodal distribution in the case of negative feedbacks) relies on their being sufficiently large amounts of initial variation in behaviour. In the current model, that initial variation is caused by relatively low cue reliabilities, which promote variation in beliefs about the environmental condition and thus initial specialization choices. Environmental uncertainty can thus be thought of as the spark that spurs the feedback engine into channeling individuals along different developmental trajectories. Indeed, in the case of maximum uncertainty (cue reliability = 0.5), positive versus negative feedbacks produce the starkest contrasts in patterns of individuality (electronic supplementary material, Appendix 3, figure A3.1; intriguingly, these patterns resemble conservative (in the case of concave fitness functions) and diversified (for convex fitness functions) bet-hedging strategies [51,52]). Environmental uncertainty, however, is only one of a range of possible causes of initial differences in behaviour [53,54]: it will be useful to explore to what extent alternative mechanisms for generating initial differences—such as negative frequency dependence [8,55] or transgenerational plasticity [56,57]—provide similar fodder on which feedbacks may act.

Future work should also explore how the strength of behaviour-state feedbacks in generating patterns of individuality might depend on both the dimensionality of phenotypic space and the rules governing the unfolding of development through that space. In the model we have employed, for example, the environment can only take one of two conditions, and individuals must increment one phenotypic unit in one of two phenotypic dimensions in each developmental timestep. While such two-environments/two-behavioural-options models are extremely common and have been powerful tools to prime intuition and produce insights in a large range of topics throughout behavioural ecology, one important next step will be to investigate the emergence of feedbacks associated with more complex behavioural traits in more graded environments. Furthermore, the accrual of ‘state’ (i.e. specialization increments) being incremental and irreversible obviously fails to capture the diversity of state variables or the ways in which state may be altered. Thus, it will be important to develop a new generation of evolutionary models that incorporate more developmental realism [58] such as richer phenotypic spaces and more diverse developmental rules for traversing those spaces. Such models are likely to broaden our understanding of one of the most intriguing aspects of organismic life: the complex web of ecological and evolutionary forces underlying developmental trajectories and its consequences for the emergence of individuality.

## Data Availability

All model code is publicly accessible on Github (https://github.com/smehlman/Developmental_feedbacks) and has been archived within the Zenodo repository: https://doi.org/10.5281/zenodo.7299681 [59]. Further model details and results are provided in electronic supplementary material [60].
